# T129. CHARACTERISTICS OF PREMORBID FUNCTIONING IN MALE ADOLESCENTS WHO LATER SUFFERED FROM PSYCHOTIC DISORDERS

**DOI:** 10.1093/schbul/sby016.405

**Published:** 2018-04-01

**Authors:** Katya Rubinstein, Ortal Bhuknik-Atzil, Rivka Tuval-Maschiach, Eyal Fruchter, Avi Reichenberg, Mark Weiser

**Affiliations:** 1 Sheba Medical Center; 2 Bar-Ilan University; 3 Israeli Defense Forces; 4 Icahn School of Medicine at Mount Sinai

## Abstract

**Background:**

Previous research has shown that people with psychotic disorders have impaired functioning prior to the onset of the illness. The main goal of the proposed study was to deepen understanding of the characteristics of premorbid impairment in persons later diagnosed with psychotic disorders.

**Methods:**

We examined unique premorbid data from IDF archives, including narrative summaries of pre-induction interviews of 17 years-old adolescents. This nested case-controlled study sample included two groups: 168 male adolescents who were later hospitalized for psychotic disorders, and 168 matched control subjects who were not diagnosed with psychotic illness during their military service. All subjects underwent pre-induction assessments between 2001 – 2010. The data were analyzed using mixed-method analysis, combining qualitative and quantitative research methods, in order to present an integrated characterization of pre-morbid functioning of future cases, compared to controls. Themes that arose from qualitative analyses, were conceptually divided into life conditions (for instance, death of a close person), and personal characteristics (i. e., mature, responsible). Each theme group was clustered into factors using categorical principal components analysis (CATPCA). Between-group comparisons on the identified factors were performed. Afterwards, the factors that were identified as significantly different in between-group comparisons, were included in a classification tree analyses to examine possible predictors of outcome.

**Results:**

The analyses identified 5 factors in the “states” category: adaptation difficulties, negative family environment, suicidal thoughts and experience, medical conditions, and loss and instability in the family. In the “traits” category, 5 additional factors were identified: high-functioning, unpleasant interpersonal impression, interpersonal trust issues, strange impression, and low social skills. Future psychotic disorder patients, compared with matched controls, showed more premorbid adaptation difficulties. Their family environments were characterized with more serious medical or psychiatric conditions, experience of loss and instability, as well as family disruption and violence. Their personality traits were characterized by low interpersonal skills, while controls were described as more “high-functioning”.

**Discussion:**

The current study partially replicated previously published findings and provided detailed description of the characteristics of environment, functioning and personal traits of people who experienced first outbreak of psychotic disorder, in the years before the outcome. Unlike most of the studies that focused on premorbid period, the current study used unique premorbid data and a combination of in-depth qualitative analyses, performed blinded to outcome, and novel machine learning techniques.

